# MexXY multidrug efflux system of *Pseudomonas aeruginosa*

**DOI:** 10.3389/fmicb.2012.00408

**Published:** 2012-11-28

**Authors:** Yuji Morita, Junko Tomida, Yoshiaki Kawamura

**Affiliations:** Department of Microbiology, School of Pharmacy, Aichi Gakuin UniversityNagoya, Japan

**Keywords:** aminoglycoside resistance, *Pseudomonas aeruginosa*, efflux, MexXY, PA5471, OprA

## Abstract

Anti-pseudomonas aminoglycosides, such as amikacin and tobramycin, are used in the treatment of *Pseudomonas aeruginosa* infections. However, their use is linked to the development of resistance. During the last decade, the MexXY multidrug efflux system has been comprehensively studied, and numerous reports of laboratory and clinical isolates have been published. This system has been increasingly recognized as one of the primary determinants of aminoglycoside resistance in *P. aeruginosa*. In *P. aeruginosa* cystic fibrosis isolates, upregulation of the pump is considered the most common mechanism of aminoglycoside resistance. Non-fermentative Gram-negative pathogens possessing very close MexXY orthologs such as *Achromobacter xylosoxidans *and various *Burkholderia* species (e.g., *Burkholderia pseudomallei* and *B. cepacia* complexes), but not *B. gladioli*, are intrinsically resistant to aminoglycosides. Here, we summarize the properties (e.g., discovery, mechanism, gene expression, clinical significance) of the *P. aeruginosa* MexXY pump and other aminoglycoside efflux pumps such as AcrD of *Escherichia coli*, AmrAB-OprA of *B. pseudomallei*, and AdeABC of *Acinetobacter baumannii*. MexXY inducibility of the PA5471 gene product, which is dependent on ribosome inhibition or oxidative stress, is noteworthy. Moreover, the discovery of the cognate outer membrane component (OprA) of MexXY in the multidrug-resistant clinical isolate PA7, serotype O12 deserves special attention.

## INTRODUCTION

*Pseudomonas aeruginosa* has been recognized as an increasingly important and worrisome species in health care-associated infections (Poole, 2011). This bacterium possesses intrinsic resistance to many antimicrobials because of the low permeability of its outer membrane barrier and the presence of multidrug efflux transporters (Nikaido, 1994; Hancock, 1998). Although fluoroquinolones (e.g., ciprofloxacin), broad-spectrum β-lactams (e.g., imipenem), and anti-pseudomonas aminoglycosides (e.g., amikacin) are often available for treatment, *P. aeruginosa* readily acquires resistance to these anti-pseudomonas agents via chromosomal mutations and lateral gene transfer (Poole, 2011). The emergence and spread of multidrug-, extensive drug-, and pandrug-resistant *P. aeruginosa* infections is of great concern as very few agents are effective against these strains (Fischbach and Walsh, 2009; Poole, 2011). The problem of increasing antimicrobial resistance is even more threatening when considering the very limited number of new antimicrobial agents in development (Fischbach and Walsh, 2009). In particular, the prospects of finding new antibiotics for Gram-negative pathogens are poor because of the low permeability of their outer membrane barriers and the presence of multidrug efflux transporters (Fischbach and Walsh, 2009). To combat these bacteria efficiently, it is necessary to understand the molecular basis of the efflux mechanisms involved in limiting the intracellular (or periplasmic) concentration of many antibiotics (Nikaido and Pages, 2012).

The most clinically relevant multidrug efflux systems in Gram-negative bacteria are those of the resistance-nodulation-cell division (RND) family (Poole, 2004, 2012; Piddock, 2006; Lister et al., 2009).* P. aeruginosa* expresses several RND-type multidrug efflux systems, of which MexAB-OprM, MexCD-OprJ, MexEF-OprN, and MexXY are significant determinants of multidrug resistance in laboratory and clinical isolates (Poole, 2004, 2012; Piddock, 2006; Lister et al., 2009). These pumps are three-component systems comprising antiporters of the RND family driven by proton motive force (MexB, MexD, MexF, and MexY), outer membrane factors (OMF; OprM, OprJ, and OprN), and periplasmic membrane fusion proteins (MFP; MexA, MexC, MexF, and MexX; Piddock, 2006; Lister et al., 2009). These pumps probably function in a similar manner with AcrAB-TolC, which is the best-studied RND-type multidrug pump of *Escherichia* *coli* (Nakashima et al., 2011; Nikaido, 2011).

Among them, the MexXY system is intriguing in that it is a significant determinant of aminoglycoside resistance only in *P. aeruginosa*, with numerous reports of clinical isolates during the last decade (Nikaido and Pages, 2012; Poole, 2012). Relatively few bacterial drug efflux systems are known to accommodate aminoglycosides (Poole, 2012). In addition to MexXY, AmrAB-OprA of *B. pseudomallei* is noteworthy for its contribution to this organism’s intrinsic aminoglycoside resistance, while AdeABC of *Acinetobacter baumannii* is implicated in acquired aminoglycoside resistance (Poole, 2012). The upregulation of the MexXY pump is considered the most common mechanism of resistance (Armstrong and Miller, 2010) and appears to be the major determinant of aminoglycoside resistance in cystic fibrosis (CF) lung isolates of *P. aeruginosa* (Poole, 2011). Here, we summarize the properties of these pumps and discuss how to combat efflux-mediated aminoglycoside resistance.

## PRE-MexXY DISCOVERY ERA: RND MULTIDRUG EFFLUX PUMPS AS DETERMINANTS OF RESISTANT TO A WIDE RANGE OF ANTIMICROBIALS, BUT NOT AMINOGLYCOSIDES

In 1993, the first RND-type multidrug efflux system of *P. aeruginosa*, MexAB-OprM (OprM was called OprK at that time), was discovered at approximately the same time as the AcrAB (AcrB was called AcrE at that time) system of *E. coli *(Poole et al., 1993b). It was the first genetic evidence that an efflux operon was involved in multiple antibiotic resistance in *P. aeruginosa *(Poole et al., 1993b). The following year, the efflux activity of tetracycline, chloramphenicol, norfloxacin, and benzylpenicillin was shown using antibiotic accumulation assays in intact cells, which was the first biochemical evidence of the role of efflux in intrinsic multidrug resistance in *P. aeruginosa* (Li et al., 1994a,b; Li et al., 1995). Taken together, the MexAB-OprM system was shown to contribute to the intrinsic resistance of *P. aeruginosa *to a wide range of antimicrobial compounds including fluoroquinolones, tetracycline, chloramphenicol, and β-lactams such as carbenicillin (Poole et al., 1993b; Li et al., 1995). Its homologs (MexCD-OprJ and MexEF-OprN) were then discovered as determinants of *nfxB* and *nfxC* fluoroquinolone-resistant (e.g., norfloxacin) mutants from *P. aeruginosa* (Poole et al., 1996; Kohler et al., 1997). Incidentally, these two pumps are not expressed in normal laboratory growth conditions, but are induced under some conditions in wild-type *P. aeruginosa* (e.g., Morita et al., 2003; Fetar et al., 2011). An unidentified efflux system that requires OprM was shown to contribute to resistance to quinolones and cephalosporins, such as cefpirome, erythromycin, and tetracycline, but not β-lactams, such as cefoperazone and carbenicillin, in the *P. aeruginosa* PAO1 background (Zhao et al., 1998). More details on these three pumps can be found in recent reviews (e.g., Li and Nikaido, 2004, 2009; Lister et al., 2009).

In those days, RND multidrug efflux systems such as MexAB-OprM and AcrAB-TolC, which can handle a wide variety of drugs that appear to contain hydrophobic domains of significant sizes (Nikaido, 1996), were considered to be similar to the P-glycoprotein multidrug efflux pump of mammalian cells, which extrudes not only basic compounds but also neutral and weakly acidic compounds (Nikaido, 1994). However, there was no evidence for the efflux of aminoglycosides, which are very hydrophilic compounds, among the antibiotics used to treat *P. aeruginosa *infections (Li et al., 1994a; Nikaido, 1996).

## MexXY SYSTEM OF *P. aeruginosa* WAS IDENTIFIED BY THREE DIFFERENT GROUPS

The MexXY system was discovered in 1999 in Japan as the fourth RND-type multidrug efflux system of *P. aeruginosa* PAO1 (Mine et al., 1999). This system was functionally expressed and conferred resistance to fluoroquinolones, tetracycline, erythromycin, etc. in the *E. coli* KAM3 mutant (Morita et al., 1998) lacking the *acrB* gene, which is an RND transporter component of the major multidrug efflux pump (AcrAB-TolC) in *E. coli*. Interestingly, unlike the other three already known systems, no open-reading frame encoding the outer membrane component, such as OprM, was found in the region downstream from the *mexY* gene (Mine et al., 1999). However, this system was found to function cooperatively with OprM of *P. aeruginosa* and TolC of *E. coli *(Mine et al., 1999).

Nine months after the discovery described above, a French group showed that MexXY was involved in the natural resistance of *P. aeruginosa* PAO1 to aminoglycosides as well as tetracycline and erythromycin (Aires et al., 1999). Although the overexpression of MexXY increased the level of resistance to fluoroquinolones in *P. aeruginosa *PAO1 cells, disruption of *mexXY* from PAO1 had no detectable effect on susceptibility to these agents (Aires et al., 1999). *mexZ*, which is located upstream of but transcribed separately from *mexXY*, was identified (Aires et al., 1999). Its product, MexZ, contains a helix-turn-helix motif, which is characteristic of DNA-binding proteins, at its N-terminus, similar to the repressors of RND-type multidrug efflux genes (e.g., AcrR, a repressor of *acrAB* in *E. coli*), supporting the notion that *mexZ* negatively controls the expression of the operon (Aires et al., 1999).

The following month (10 months after the first discovery), a group in the USA showed that MexXY, which they called AmrAB, was an aminoglycoside impermeability factor in spontaneous aminoglycoside-resistant mutants of the impermeability phenotype from *P. aeruginosa* PAO1 (Westbrock-Wadman et al., 1999). Interestingly, a dramatic decrease in the amount of OprM was observed in the mutants compared to wild-type PAO1, indicating that OprM is unlikely to be the outer membrane component associated with this efflux system in the mutants (Westbrock-Wadman et al., 1999). In addition, MexXY was shown to be upregulated in clinical *P. aeruginosa* isolates displaying aminoglycoside impermeability, suggesting that the pump is a clinically relevant mechanism of aminoglycoside resistance in *P. aeruginosa *(Westbrock-Wadman et al., 1999).

The following year, the complete genomic sequence of *P. aeruginosa* strain PAO1 (PAO1-UW) was published in *Nature* (Stover et al., 2000). Although the locus IDs PA2019-18 of the PAO1-UW genome sequence correspond to the *mexXY* genes, the nucleotide sequences of PA2019-18 were not identical with those of previously published *mexXY *(Aires et al., 1999; Mine et al., 1999) findings. This is probably because the DNA sequencing technology at that time was unable to analyze GC-rich bacteria such as *P. aeruginosa *(66–67% GC content; Winsor et al., 2011). Therefore, we analyzed MexXY using the nucleotide sequences from the PAO1-UW complete genome (Winsor et al., 2011) because we live in the post-genome era.

## STRUCTURE AND FUNCTION OF MexY

The RND components of RND-type tripartite multidrug efflux pumps determine substrate specificity (e.g., Srikumar et al., 1997; Eda et al., 2003); therefore, we focused on the structure and function of MexY rather than MexX or OprM. Very recently, the crystal structure of the RND-type multidrug efflux pump AcrB of *E. coli* revealed the presence of two discrete, high-volume multisite binding pockets that contribute to the remarkably broad substrate recognition of AcrB and its homologs (Nakashima et al., 2011). Although we basically assume that MexY pumps out antimicrobials in a similar manner as AcrB, it will be intriguing to uncover the molecular basis of how MexY accommodates aminoglycosides because they are strongly hydrophilic molecules that are completely different from the relatively hydrophobic compounds (e.g., minocycline, doxorubicin, rifampicin, and erythromycin) used as substrates of AcrB (Nakashima et al., 2011).

Generally speaking, the function of a transporter (e.g., substrate specificity and energy coupling) should be determined by its time course efflux assay and evaluated using kinetic constants (e.g., Yerushalmi et al., 1995; Edgar and Bibi, 1997; Mine et al., 1998; Morita et al., 2000). However, it is difficult to conduct such an assessment in a small bacteriology laboratory. The reconstitution of proteoliposomes revealed that AcrB, AcrD, and MdtBC of *E. coli* were H^+^/drug antiporters (Zgurskaya and Nikaido, 1999; Aires and Nikaido, 2005; Kim et al., 2010), and we assume that MexY pumps out antimicrobials coupled with the same energy. In addition, five charged and polar amino acid residues that are involved in the proton translocation pathway are conserved between MexY and AcrB of *E. coli *(Takatsuka and Nikaido, 2006). Unfortunately, the purification, reconstitution, and characterization of the MexXY pump remain to be established, and the energy coupling and substrate specificity of MexXY has not been examined through its efflux activity. On the other hand, the MexXY-mediated energy-dependent efflux activity of ethidium (Mine et al., 1999), aminoglycosides (Aires et al., 1999; Vogne et al., 2004), tetracycline (Aires et al., 1999), Ala-Nap (MC-005,556) (Mao et al., 2001), and fluorescein-di-β-D-galactopyranoside (Matsumoto et al., 2011) has been measured in whole cells.

It is conventional to use differences in minimum inhibitory concentrations (MICs) between bacterial cells with and without a multidrug efflux transporter to estimate substrate specificity (e.g., Nishino and Yamaguchi, 2001; Nishino et al., 2003). Although the comparison of MICs can sometimes be a misleading indicator of pump function (e.g., Nagano and Nikaido, 2009), it can still indicate the possible clinical relevance of a pump (e.g., Poole, 2004, 2012; Piddock, 2006). Mutant strains lacking major multidrug efflux pump(s) have been used to determine substrate specificity (e.g., Nishino and Yamaguchi, 2001; Morita et al., 2001b). The substrate specificity of MexXY-OprM was determined using a mutant from PAO1 that overproduced MexXY-OprM, but not MexAB (and AmpC in the case of β-lactams), and were compared with a mutant lacking MexXY/MexAB-OprM (and AmpC in the case of β-lactams; Masuda et al., 2000b). MexXY-OprM-mediated resistance was then observed for quinolones, macrolides, tetracyclines, aminoglycosides, chloramphenicol, lincomycin, and most β-lactams, but not for novobiocin, polymyxin B, and some β-lactams (carbenicillin, sulbenicillin, cefsulodin, ceftazidime, oxacephem, imipenem, and aztreonam) among a wide variety of antimicrobial agents (Masuda et al., 2000b). In conclusion, MexXY-OprM is a multidrug efflux transporter whose specificity is extraordinary broad, but different compared with MexAB-OprM, MexCD-OprJ, MexEF-OprN, and other RND efflux transporters in *P. aeruginosa*. In addition, MexXY-OprM was the only pump to mediate aminoglycoside resistance and was thus considered to recognize aminoglycosides as substrates (Masuda et al., 2000b).

Basic local alignment search tool (BLAST) analysis showed that MexY was highly conserved in *P. aeruginosa* strains: more than 99% (99%) identity (positive) for most strains and 97% (98%) identity (positive) for PA7 (Morita et al., 2012). There was no functional difference between the MexYs of PAO1 and PA7 when they were expressed in either *E. coli* or *P. aeruginosa* (Morita et al., 2012). MexY was more similar [70–73% (83–86%) identity (positive)] to orthologs of *B. pseudomallei *and various *B. cepacia *complexes than other RND pumps of *P. aeruginosa *and other* Pseudomonas *species(Morita et al., 2012). These *Burkholderia* species, except for *B. mallei*, are intrinsically resistant to aminoglycosides (e.g., Kenny et al., 1999; Thibault et al., 2004; Vermis et al., 2003; Jassem et al., 2011). *B. gladioli *is also known to be involved in human infections (Segonds et al., 2009); however, no MexY (AmrB) ortholog exists in *B. gladioli *BSR3 (Seo et al., 2011), consistent with the fact that all isolates tested were susceptible to aminoglycosides (Segonds et al., 2009). Interestingly, the most similar functional ortholog to MexY exists in *Achromobacter xylosoxidans* and has a 74% identity (86% positive); this pump was named AxyY in strain AXX-A (Bador et al., 2012). *A. xylosoxidans* is also an opportunistic human pathogen capable of causing a wide range of infections (Glupczynski et al., 1988; Bador et al., 2011). Most *A. xylosoxidans* clinical isolates were resistant to the tested aminoglycosides, including amikacin (Ngeow and Puthucheary, 1985; Glupczynski et al., 1988). The AxyY pump contributes to aminoglycoside resistance in a similar manner to MexY and AmrBs (Bador et al., 2012).

**FIGURE 1:**
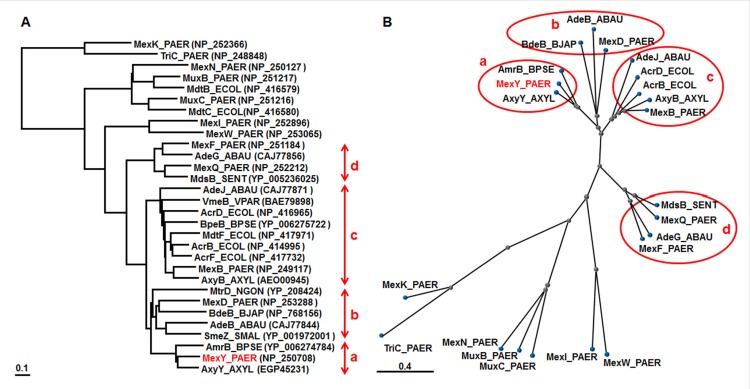
**Phylogenetic trees for representative RND transporters**. According to the COBALT program, the trees were constructed using the Fast evolution method and rendered with **(A)** Rectangle and **(B)** Radical. Protein names are abbreviated; for example, “MexY_PAER” stands for “MexY of *P. aeruginosa*.”Accession numbers are shown in parentheses. The four branches shown in red are: (a) MexY, (b) MexD, (c) MexB, and (d) MexF.

COBALT analysis is a multiple sequence alignment tool for finding a collection of pairwise constraints. Such constraints are derived from data of the conserved domain database, protein motif database, and sequence similarity of RND pumps (Papadopoulos and Agarwala, 2007), including all pumps from *P. aeruginosa* PAO1-UW and *E. coli *K12 (MG1655). The exception is heavy metal efflux pumps, which are characterized by their relationships. Therefore, we focused on the four branches containing the four Mex pumps in *P. aeruginosa* (**Figure 1**). The MexY branch is located next to the MexD branch and includes the AmrBs of *Burkholderia *species (e.g., Mima and Schweizer, 2010) and AxyY of *A. xylosoxidans *(Bador et al., 2012). The MexD branch includes the AdeB pump of *A. baumannii *(Magnet et al., 2001), MtrD of *Neisseria gonorrhoeae *(Hagman et al., 1997),and BdeB of *Bradyrhizobium japonicum *(Lindemann et al., 2010). The SmeZ pump of *S. maltophilia*, which can mediate aminoglycoside resistance (Crossman et al., 2008), also belongs to the MexD branch. Many pumps in the MexY/MexD branches can mediate aminoglycoside resistance (e.g., Magnet et al., 2001; Crossman et al., 2008; Lindemann et al., 2010; Mima and Schweizer, 2010), which hints at the structure-function relationship of pumps involved in aminoglycoside resistance. MexB is located in the branch that contains the AcrB/D/F and MdtF pumps of *E. coli *(Nishino and Yamaguchi, 2001; Nishino et al., 2003), AdeJ of *A. baumannii *(Damier-Piolle et al., 2008), BpeB of *B. pseudomallei *(Mima and Schweizer, 2010), AxyB of *A. xylosoxidans *(Bador et al., 2011), and VmeB of *Vibrio parahaemolyticus *(Matsuo et al., 2007). Among them, some pumps (e.g., AcrD and MexB) were reported to be involved in aminoglycoside resistance under some conditions (Li et al., 2003; Aires and Nikaido, 2005). The MexF branch includes AdeG of *A. baumannii* (Coyne et al., 2010) and MdsB of *Salmonella enterica *(Nishino et al., 2006).

Substrate specificity is determined almost entirely by the periplasmic domain (i.e., two large extramembrane loops that largely protrude toward the periplasmic space) of MexY (and MexB; Eda et al., 2003). MexY_F1018L_ (the F1018L mutation is located in TMS-12 of MexY base on the structure of AcrB; Murakami et al., 2002), enhanced the function of MexY, presumably by increasing the efflux of aminoglycosides, cefepime, and fluoroquinolones, which was the first example of an improved efflux pump *in vivo *(Vettoretti et al., 2009b).

## GENE EXPRESSION OF THE MexXY SYSTEM

MexXY was shown to be induced by sub-inhibitory concentrations of tetracycline, erythromycin, aminoglycosides, tigecycline, and LMB415 (a peptide deformylase inhibitor), but not ofloxacin in *P. aeruginosa* PAO1 (Masuda et al., 2000a; Dean et al., 2003; Caughlan et al., 2009). Moreover, ofloxacin and cefpirome were also shown to be inducers, but only in a PAO1 mutant lacking MexAB (and AmpC in the case of cefpirome; Masuda et al., 2000a,b). MexZ was shown to bind an inverted repeat region located in the *mexZ*-*mexX* intergenic region directly as a homodimer, which encompasses the putative *mexXY* promoter, but the inducers failed to alter the MexZ-operator interactions (Matsuo et al., 2004). The crystal structure of MexZ has since been solved (Alguel et al., 2010). Induction of the MexXY efflux pump in *P. aeruginosa* PAO1 was shown to be dependent on drug–ribosome interactions (Jeannot et al., 2005), and the pump remained inducible, but to a lesser degree, by ribosomal inhibitors, even in the *mexZ* mutant (Jeannot et al., 2005). These data demonstrate the physiological interactions between MexXY and the ribosome and are suggestive of an alternative function for MexXY beyond the efflux of antibiotics (Jeannot et al., 2005). Microarray analysis showed that *mexXY* were the most highly upregulated genes in *P. aeruginosa* PAO1 after 4 h of interaction with primary normal human airway epithelial cells (Frisk et al., 2004) and in response to sub-inhibitory concentrations of tobramycin under normal aerobic conditions, but not under lethal aerobic conditions or anaerobic conditions (Kindrachuk et al., 2011).

The antibiotic inducibility of the MexXY multidrug efflux system of *P. aeruginosa* was shown to be involved in the modulation of MexZ activity by the antibiotic-inducible PA5471 gene product (Morita et al., 2006; **Table 1**). PA5471 encodes a predicted product of 43.5 kDa, which was identified as a hypothetical protein conserved between bacteria and archaea, and is a representative of the uncharacterized protein family UPF0027 in the Pfam protein families database (Morita et al., 2006) or the PRK09588 cluster in ProtClustDB (NCBI Protein Clusters Database; Klimke et al., 2009). Recently, it was demonstrated that RctB of *E. coli*, which is related to members of this family, is a novel RNA ligase and functions as a *bona fide* RNA repair protein *in vivo *(Tanaka and Shuman, 2011). PA5471 is found upstream of and in a possible operon with an open-reading frame dubbed PA5470; RT-PCR confirmed both the drug inducibility of PA5470 and its expression from a polycistronic message that also contains PA5471 (Morita et al., 2006). PA5470 is predicted to encode a peptide chain release factor of 22.3 kDa (Morita et al., 2006). A homolog of PA5471 from *E. coli* K12, *ykfJ* (b0235), which was, however, C-terminally truncated (approximately 1 kb; Baranov et al., 2006), was also shown to be inducible by 4-azaleucine, which is known to interfere with translation, and it too is linked to a putative peptide release factor gene (Morita et al., 2006).* P. aeruginosa* senses antibiotic-mediated ribosomal disruption and links it to PA5471 gene expression by monitoring the translation of a 13-amino-acid-leader peptide region (PA5471.1) found ~250 bp upstream of the PA5471 coding sequence on PA5471 mRNA (Morita et al., 2009). The antimicrobial-inducible PA5471 gene product has been shown to interact with the repressor MexZ and interfere with its DNA binding activity *in vitro* (Yamamoto et al., 2009), and this finding contributed to elucidating the molecular mechanisms of the MexXY induction. However, PA5471 is not sufficient for MexXY recruitment in response to antibiotic exposure, and additional antibiotic-dependent effects are needed in *P. aeruginosa *(Morita et al., 2009). Exposure to reactive oxygen species (ROS; e.g., peroxide) induces the expression of the PA5471 gene, leading to MexXY-dependent aminoglycoside resistance (Fraud and Poole, 2011). Moreover, long-term (8-day) exposure of *P. aeruginosa* to peroxide (mimicking chronic *in vivo* ROS exposure) increased the frequency of PA5471- and *mexXY*-dependentaminoglycoside resistance (Fraud and Poole, 2011). Recently, reduced (approximately twofold) expression of the *rplU-rpmA* operon (encoding the 50S ribosomal proteins L21 and L27) was shown to promote *mexXY* expression via the PA5471 gene in pan-aminoglycoside resistant mutants from PAO1 and a CF clinical isolate (Lau et al., 2012). Such expression was in the form of ribosomal protein mutations that influence *mexXY* expression, including *rplY* (encoding ribosomal protein L25; El’Garch et al., 2007) and *rplA* (encoding ribosomal protein L1; Westbrock-Wadman et al., 1999). Transcriptome profiling revealed that significantly increased expression was observed for the *mexXY* and PA5471 genes in both the PA2572 and PA2573 mutants compared with the wild-type PAO1 strain during exponential growth in Luria–Bertani media (McLaughlin et al., 2012). PA2572 encodes a putative response regulator of a two-component system required for full virulence to* Galleria mellonella* (Wax moth) and PA2573 also encodes an ophan chemotaxis sensor which seems to function in part through signal transduction involving PA2572 (McLaughlin et al., 2012).

**Table 1:** Genetic organization of aminoglycoside efflux operons of clinical significance and their regulators in non-fermentative Gram-negative pathogens.

Organism	Efflux operon	Product	Function	Regulator	
			Cognate	Other
*P. aeruginosa*	*mexXY*(*-oprA*)^a^	MexX MexY (OprA)^a^OprM	MFP RND OMF	MexZ	PA5471 ParRS
*A. xylosoxidans*	*axyXY-oprZ*	AxyX AxyY OprZ	MFP RND OMF	AxyR	
*B. pseudomallei*	*amrAB-oprA*	AmrA	MFP	AmrR	
(*B. cepacia *complex)	oprA	AmrB OprA	RND OMF		
*A. baumannii*	*adeAB*(*-adeC*)^b^	AdeA	MFP	AdeRS	?^c^
		AdeB	RND		
		(AdeC)^b^ ?^b^	OMF		

*The genetic organization of genes involved in aminoglycoside efflux and their regulation of expression is summarized. No obvious significant differences on substrate specificities were observed among the pumps. TetR-type negative regulators are encoded by genes located upstream of the operons in *P. aeruginosa*, *A. xylosoxydans*, and* B. pseudomallei*, while the AdeRS two-component regulatory proteins are encoded by genes located upstream of the *adeAB*(*-adeC*) operon.*

a *oprA* gene found in the multidrug-resistant clinical isolate PA7 and relatives, all of which are serotype O12, is absent and often OprM encoded by the *mexAB-oprM* multidrug efflux operon is associated with the MexXY component in most *P. aeruginosa* strains (Morita et al., 2012). MexXY can utilize OprA or OprM as an outer membrane channel (Morita et al., 2012).

b AdeC is not essential for AdeAB-mediated resistance (Marchand et al., 2004), suggesting that AdeAB recruits another yet unknown outer membrane protein as indicated by the question mark.

c The question mark signifies other unknown regulatory mechanism(s) involved in *adeABC *overexpression (Sun et al., 2010).

A recent study identified a gene, *parR*, encoding the response regulator of a two-component system, ParRS, which promotes either induced or constitutive *mexXY *upregulation, thereby activating the MexXY efflux system as well as OprD porin loss and lipopolysaccharide modification in a MexZ-independent manner (Muller et al., 2011). Overexpression of PaeIII, a small non-coding RNA between PA3505 and PA3536 in the genome of *P. aeruginosa* PAO1, in the stationary phase increased the expression of the *mexXY* and *mexZ *genes as well as type III secretion genes, while reducing the expression of genes for arginine metabolism (Goldberg et al., 2008).

## MexXY SYSTEM AS AN ANTIMICROBIAL RESISTANCE DETERMINANT IN *P. aeruginosa*

*Pseudomonas aeruginosa* shows intrinsic resistance against many antimicrobials because of the low permeability of its outer membrane and the presence of efflux systems (Nikaido, 1994; Hancock, 1998). MexXY was shown to be involved in natural resistance to aminoglycosides, tetracycline, tigecycline, erythromycin, and LBM415 in *P. aeruginosa* PAO1 (Aires et al., 1999; Masuda et al., 2000a; Morita et al., 2001a; Dean et al., 2003; Caughlan et al., 2009). MexXY was also shown to be necessary for the adaptive resistance of *P. aeruginosa* PAO1 to aminoglycosides (Hocquet et al., 2003). It is of note that MexXY is the only pump of the 12 identified RND systems that mediates aminoglycoside resistance in *P. aeruginosa* PAO1 (Poole, 2011). The antagonism of aminoglycosides by the divalent cations Mg^2+^ and Ca^2+^ is well documented (Medeiros et al., 1971), and culture in cation-adjusted Mueller–Hinton broth is recommended as a susceptibility test to ensure acceptable results when *P. aeruginosa* isolates are tested (Barry et al., 1992). MexXY was shown to be required for the antagonism of aminoglycosides by divalent cations in *P. aeruginosa *PAO1 (Mao et al., 2001). Although Phe-Arg-β-naphthylamide (PAβN, MC-207,110) is known as a non-specific inhibitor against RND-type multidrug efflux pumps (Lomovskaya et al., 2001), this inhibitor, as observed for divalent cations, antagonized the activity of aminoglycosides (amikacin and netilmicin) in a MexXY-dependent manner, even though it also inhibited MexXY-dependent fluoroquinolone (levofloxacin) resistance (Mao et al., 2001). Conversely, PAβN inhibited MexXY-mediated aminoglycoside (gentamicin) resistance (Mesaros et al., 2007). The reason for the discrepancy between these two results remains unknown. Increased susceptibility to aminoglycosides in *nfxB* mutants, which upregulate *mexCD-oprJ *expression, was correlated with increased resistance to fluoroquinolones and some β-lactams, such as cefepime, concomitant with a higher susceptibility to aminoglycosides and some β-lactams, such as ticarcillin, aztreonam, and imipenem. This was shown to be partly due to the impaired activity of MexXY-OprM because of major changes in cell physiology, but not the expression/production of *mexY*/MexY and *oprM*/OprM (Jeannot et al., 2008; Mulet et al., 2011). The increased susceptibility to aminoglycosides in MexEF-OprN-overproducing *nfxC* mutants was also observed, apparently owing to impairment of the MexXY system (Sobel et al., 2005). *mexXY* expression (and so MexXY-mediated resistance) was independent of the AmgRS two-component system in which mutations enhanced aminoglycoside action to control an adaptive response to membrane stress (Lee et al., 2009).

Multidrug resistant *P. aeruginosa* clinical isolates have often been reported to be MexXY overproducers (e.g., Llanes et al., 2004, 2006; Wolter et al., 2004; Deplano et al., 2005; Henrichfreise et al., 2007; Hocquet et al., 2007; Maniati et al., 2007; Vettoretti et al., 2009a; Beaudoin et al., 2010; Xavier et al., 2010; Fehlberg et al., 2012; Pasca et al., 2012). Time series analysis (January 1999 to January 2005) revealed a significant relationship between antibiotic use (aminoglycosides, fluoroquinolones, and cefepime, but not carbapenems) and the incidence of MexXY-overproducing *P. aeruginosa* in a French hospital (Hocquet et al., 2008). MexXY (*n* = 39) and MexAB (*n* = 31) were the most frequently overproduced pumps in 85 non-CF *P. aeruginosa* strains with low-level ciprofloxacin resistance (MICs ranging from 0.25 to 2 µg/mL, which are still susceptible or intermediate according to the CLSI breakpoints; Llanes et al., 2011). A large proportion of the strains were MexXY overproducers in genotypically distinct *P. aeruginosa* clinical isolates that were less susceptible to cefepime than to ceftazidime, and these were identified in Europe (Hocquet et al., 2006; Pena et al., 2009; Campo Esquisabel et al., 2011) and the USA (Laohavaleeson et al., 2008). In contrast, both cefepime and ceftazidime are potent β-lactam antibiotics with similar MICs (1–2 µg/mL) for wild-type *P. aeruginosa* strains. Moreover, ceftobiprole, similar to cefepime, selected MexXY overproducers in clinical studies (Baum et al., 2009). Actually, a single step MexXY overproducer was selected *in vitro* by cefepime and ceftobiprole, but not ceftazidime (Queenan et al., 2010). MexXY contributed very significantly to the development of high-level (100–1000 µg/mL MIC) aminoglycoside resistance via a combination of aminoglycoside-modifying enzymes (AMEs) in multidrug resistant *P. aeruginosa* non-CF clinical isolates (Morita et al., 2012). However, AMEs are common determinants of aminoglycoside resistance in *P. aeruginosa*,except for CF isolates (Poole, 2011). In clinical CF isolates, MexXY has been primarily implicated in pan-aminoglycoside resistance (e.g., Sobel et al., 2003; Vogne et al., 2004; Islam et al., 2004, 2009). MexXY was also shown to be necessary in subpopulations of *P. aeruginosa *CF isolates that are hypersensitive to ticarcillin (called Tic^hs^; Vettoretti et al., 2009b). *mexZ* was shown to be one of the most frequently mutated genes during chronic infection by *P. aeruginosa* in CF patients (Smith et al., 2006; Feliziani et al., 2010). However, a number of studies highlighted the absence of mutations in *mexZ* or the *mexXY* promoter region in MexXY-overproducing *P. aeruginosa* CF isolates (Sobel et al., 2003; Vogne et al., 2004; Islam et al., 2009). To date, three kinds of mutants (*agrZ*, *agrW1*, and *agrW2*) have been recognized as MexXY overproducers as a result of genetic mechanisms: mutants with impaired binding or unbinding of MexZ due to alterations in the *mexZ* or *mexZ-mexX* intergenic region (type *agrZ*); mutants with impaired protein synthesis (type *agrW1*); and mutants with alterations in *parRS* (type *agrW2*; de Bentzmann and Plesiat, 2011). Oxidative stress, a component of the host’s immune system in the CF lung, induced *mexXY *expression via PA5471 and promoted aminoglycoside resistance (Fraud and Poole, 2011). Under conditions of oxidative stress, *P. aeruginosa* can develop aminoglycoside resistance, even in the absence of aminoglycosides (Poole, 2012). It is also very plausible that the routine use of aminoglycosides (e.g., tobramycin) might simply select for MexXY-overproducing *P. aeruginosa* in theCF lung (Smith et al., 2006).

Although it is obvious thatMexXY is one of the determinants of antimicrobial resistance in *P. aeruginosa *in the clinical setting (Poole, 2011), only a few reports have assessed the *in vivo* impact of the MexXY system on antibiotic therapy for *P. aeruginosa *infections (e.g., Martha et al., 2006).

## COGNATE OUTER-MEMBRANE COMPONENT OprA OF THE MexXY PUMP IS FOUND IN SEROTYPE O12 BUT IS LOST IN OTHERS

**FIGURE 2:**
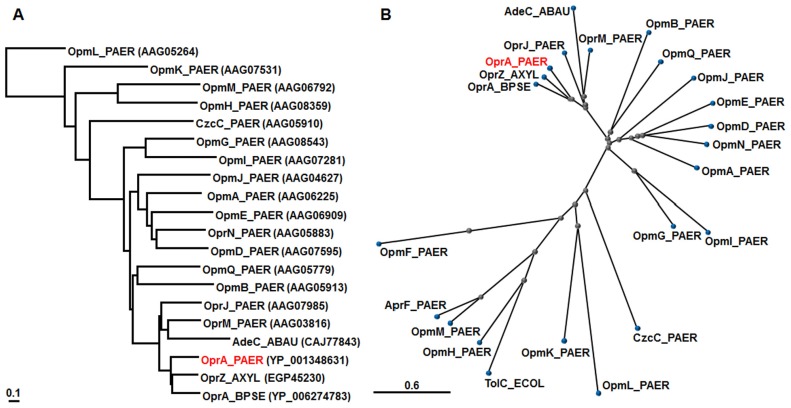
**Phylogenetic trees for representative OMPs**. According to the COBALT program, the trees were constructed using the Fast evolution method and rendered with **(A)** Rectangle and **(B)** Radical. Protein names are abbreviated; for example, “OprA_PAER” stands for “OprA of *P. aeruginosa*.” Accession numbers are shown in parentheses.

The *mexXY* operon lacks a gene coding for the outer membrane protein in *P. aeruginosa* PAO1 (Mine et al., 1999). OprM is necessary for the function of MexXY and MexAB in *P. aeruginosa* PAO1 (Aires et al., 1999; Masuda et al., 2000a; Morita et al., 2001a), although overproduced OpmB (PA2525) can function as an outer membrane component of MexXY, MexAB, and MexCD (Murata et al., 2002). Intriguingly, the multidrug resistant taxonomic outlier *P. aeruginosa* PA7 possesses a unique gene (*oprA*) downstream of *mexXY* encoding an outer membrane channel that is absent in most *P. aeruginosa* strains (Roy et al., 2010). MexXY in this strain utilizes either the OprA or OprM outer membrane channel (Morita et al., 2012; Table 1). While OprM is functional with both MexXY and MexAB, OprA did not associate as strongly with MexAB as it did with MexXY (Morita et al., 2012). We compared the OprA of *P. aeruginosa* PA7 with the OprM family (Remans et al., 2010) from *P. aeruginosa* PAO1 as well as TolC of *E. coli *K12 (Mine et al., 1999), OprA of *B. pseudomallei* 1026b (Mima and Schweizer, 2010), OprZ of *A. xylosoxidans *AXX-A (Bador et al., 2012),and AdeC *of A. baumannii* AYE (Magnet et al., 2001; **Figure 2**). COBALT analysis showed that OprA of *P. aeruginosa* PA7 and its close orthologs (OprA of *B. pseudomallei* 1026b and OprZ of *A. xylosoxidans* AXX-A) is located close to OprJ and is followed by OprM of the OprM outer membrane family of *P. aeruginosa *PAO1 (**Figure 2**).

Interestingly, a small portion of the *oprA* gene immediately downstream of the *mexY *gene in PAO1 was identified, suggesting that non-PA7 *P. aeruginosa* strains might have possessed, but lost, the intact *mexXY-oprA* efflux pump locus (Morita et al., 2012; Table 1). Consistent with this, the majority of a panel of serotype strains possessed the truncated *oprA*, but the serotype O12 isolate had an intact *mexXY-oprA* locus, similar to PA7 and the related strain DSM 1128 (Morita et al., 2012). O12 is a predominant serotype associated with multidrug resistance to a number of antibiotic classes, including aminoglycosides and β-lactams, although it represents a minor serotype in the environment (Pirnay et al., 2009; Roy et al., 2010). O12 might be more dominant due, in part, to the presence of *oprA* in hospitals in which antimicrobials promoting MexXY-OprA-mediated multidrug resistance, such as aminoglycosides, were used. *P. aeruginosa* PA7 isolated before 1984 from a wound infection in Buenos Aires, Argentina (Pirnay et al., 2009; Roy et al., 2010), might also have acquired multidrug resistance via the heavy use of antibiotics, including gentamicin or tobramycin, to treat wounds at that time. Apparently, a slightly increased resistance (two- to fourfold) to amikacin, ciprofloxacin, and cefpirome was shown in the presence and absence of *oprA* (Morita et al., 2012). Such a small difference might be significant during antibiotic treatment or in the presence of sub-inhibitory concentrations of antibiotics.

## AcrD IS AN AMINOGLYCOSIDE EFFLUX PUMP THAT IS THE MOST SIMILAR TO MexY AMONG THE RND PUMPS IN *E. coli* K12

AcrD has the highest similarity score at the amino acid level to MexY of the *E. coli* K12 RND pumps and was shown to be an aminoglycoside efflux pump as judged by MIC determination and the aminoglycoside efflux assay (Rosenberg et al., 2000). However, differences in aminoglycoside resistance (uptake) between the parent strain JC7623 and its *acrD*-deletion mutant JZM320 was possibly not limited to AcrD function because JZM320 was constructed by inserting the *tet* gene from pBR322 into *acrD* (Rosenberg et al., 2000). The increased aminoglycoside uptake might be due to not only AcrD deficiency but also to the production of an aberrant cytoplasmic membrane protein (the product of *acrD* with the inserted *tet*) and/or the tetracycline/H^+^ antiporter itself (Merlin et al., 1989a,b; Wyka and St John, 1990). While disruption of *tolC* or *acrA* did not increase the susceptibility of K12 to aminoglycosides (Rosenberg et al., 2000), both of them were necessary for the function of *acrD *against various antimicrobials; however, no aminoglycosides were used in the study (Hirakawa et al., 2003). We do not rule out the hypothesis of Rosenberg et al. (2000) that AcrD protein can perhaps function without the participation of AcrA and TolC in the case of aminoglycoside efflux.

It is evident that purified AcrD can function as an H^+^-driven aminoglycoside efflux pump (Aires and Nikaido, 2005). Especially, strong stimulation of proton efflux was observed when aminoglycosides (e.g., streptomycin) were added to the more acidic intra-vesicular space of reconstituted AcrD proteoliposomes containing AcrA and Mg^2+^(Aires and Nikaido, 2005), indicating that AcrD captures aminoglycosides exclusively from the periplasm in *E. coli* (Nikaido, 2011). The difference in the MICs of amikacin and gentamicin between a parent strain and its in-frame *acrD*-deletion mutant or between an *acrBD*-deletion mutant and its *acrD*-overexpressing complementation mutant was approximately twofold (Elkins and Nikaido, 2002; Aires and Nikaido, 2005). There was no significant difference in kanamycin resistance in the case of an in-frame deletion (Hirakawa et al., 2003), and a twofold difference was observed in the case of overproduction (Nishino and Yamaguchi, 2001; Nishino et al., 2010). An *acrA *in-frame deletion mutant also showed an approximately twofold increased susceptibility to aminoglycosides (Aires and Nikaido, 2005). However, similar observations were not seen for AcrB (Nishino and Yamaguchi, 2001; Elkins and Nikaido, 2002; Aires and Nikaido, 2005). A comparison of the entrances of the vestibules, which are found in the central cavities (Murakami et al., 2002) of AcrD (which transports aminoglycosides) and AcrB (which does not) crystal structures, shows that this area in AcrD is in line with many more acidic residues that may attract polycationic substrates (Yu et al., 2003). Treatment with sub-inhibitory concentrations of kanamycin induced adaptive resistance to aminoglycosides, which was dependent on *acrD* (Sidhu et al., 2012). Aminoglycosides are very hydrophilic and polycationic and assumingly permeate through the porin channel in *E. coli*, unlike *P. aeruginosa* (Nikaido and Pages, 2012) in addition to so called “self-promoted” aminoglycoside uptake across the outer membrane of both of *E. coli* and *P. aeruginosa* (Hancock et al., 1991; Hancock, 1998). The MICs of aminoglycosides on *E. coli*, unlike *P. aeruginosa*,might be poor indicators of aminoglycoside efflux. There are numerous AcrD homologs in other Enterobacteriaceae(Poole, 2004). Although the AcrD of* S. enterica* serovar Typhimurium ATCC 14028s was studied comprehensively, no significant difference between the AcrDs of *E. coli *and *S. enterica *has been observed so far (Nishino et al., 2009; Horiyama et al., 2011; Yamasaki et al., 2011). Interestingly, AcrD pumps mediate resistance to the substrates of MexAB (e.g., carbenicillin, aztreonam, and novobiocin). However, AcrD pumps did not mediate resistance to the substrates of MexXY (e.g., cefpirome, erythromycin, and tetraphenylphosphonium) or shared substrates of both MexAB and MexXY (e.g., fluoroquinolone and tetracycline) when differences of the MICs were compared between a parent strain and its transformant overproducing the pump (Srikumar et al., 1997; Mine et al., 1999; Morita et al., 2001a; Nishino et al., 2003, 2009; Horiyama et al., 2011; Yamasaki et al., 2011). MexAB-OprM was also shown to contribute to aminoglycoside resistance, presumably via active efflux in the low-ionic-strength medium used in this particular study (Li et al., 2003). AcrD and the MdtABC pump were iron-regulated, induced in low-iron conditions, and export the siderophore enterobactin (Bochner et al., 2008), which reminds us that MexAB-OprM was inducible under conditions of iron limitation and compensated for a growth defect in an iron-deficient medium in the presence of the non-metabolizable iron chelator 2,2′-dipyridyl (Poole et al., 1993a,b). AcrD seems to be a functional homolog of MexB rather than MexY, as determined from substrate specificity and physiological function, consistent with the fact that phylogenetic analysis showed that AcrD is closer to MexB than to MexY (**Figure 1**).

## AmrAB-OprA IS A MULTIDRUG EFFLUX SYSTEM THAT MEDIATES AMINOGLYCOSIDE RESISTANCE IN *B. pseudomallei*

*Burkholderia pseudomallei* is the etiologic agent of melioidosis, a rare but serious disease endemic to South Asia, Northern Australia, and other parts of the tropics (Mima and Schweizer, 2010). Melioidosis is very difficult to treat because of the intrinsic resistance to many antimicrobial agents including aminoglycosides, macrolides, polymyxins, and some β-lactams (Mima and Schweizer, 2010). AmrAB-OprA was identified as an efflux determinant of resistance to aminoglycosides and macrolides in the *B. pseudomallei *1026b clinical isolate (Moore et al., 1999). This pump was actually the first to be demonstrated responsible for the aminoglycoside resistance of RND pumps in Gram-negative bacteria. The gene product of *amrR*, which is located immediately upstream and divergently transcribed from *amrAB-oprA* in *B. pseudomallei* 1026b (Moore et al., 1999), showed strong homology [60% (73%) identity (positive)] to MexZ, which acts as a transcriptional repressor of the *mexXY *operon of *P. aeruginosa* PAO1 (Matsuo et al., 2004; Alguel et al., 2010; Table 1).

While the majority of *B. pseudomallei *clinical isolates exhibit high levels of aminoglycoside and macrolide resistance, rare isolates are susceptible to these antibiotics (Simpson et al., 1999; Trunck et al., 2009). While it is noted that the resistance profile of those isolates matches that of the *amrAB-oprA* mutants (Simpson et al., 1999), it was shown experimentally that *amrAB-oprA* was missing in *B. pseudomallei* 708a, an aminoglycoside- and macrolide-susceptible clinical isolate, and this loss was associated with the deletion of >130 kb of genetic material (Trunck et al., 2009). The expression of *amrAB-oprA* increased resistance to not only aminoglycosides and macrolides but also fluoroquinolones and tetracyclines in a BpeAB-OprA pump-deficient mutant of 1026b (Mima and Schweizer, 2010). Judging from the substrate specificity and sequence similarity (Mima and Schweizer, 2010), we have no doubt that AmrAB is a functional ortholog of MexXY in *B. pseudomallei. *BpeAB-OprB of *B. pseudomallei* also reportedly mediates aminoglycoside resistance in strain KHW (Chan et al., 2004), while this pump did not confer aminoglycoside resistance in 1026b (Mima and Schweizer, 2010). In addition, the BpeB RND transporter was also shown to be closely related to MexB of *P. aeruginosa*, both functionally and phylogenetically (Mima and Schweizer, 2010), consistent with our phylogenetic analysis (**Figure 1**).

As described above, AmrB orthologs are conserved among various human pathogens belonging to *Burkholderia* species, but not *B. gladioli*. Actually, an AmrAB-OprA ortholog was shown to be a major aminoglycoside resistance contributor in *B. cenocepacia*, a member of the *B. cepacia* complex (Hamad et al., 2010).

## AdeABC IS A MULTIDRUG EFFLUX SYSTEM THAT MEDIATES AMINOGLYCOSIDE RESISTANCE IN *A. baumannii*

*Acinetobacter baumannii *is the most frequently implicated species in nosocomial infections among *Acinetobacter *spp. (Coyne et al., 2011). AdeABC was identified as an RND-type efflux pump involved in resistance to multiple antimicrobials including aminoglycosides, fluoroquinolones, tetracycline, erythromycin, cefotaxime, trimethoprim, and chloramphenicol in *A. baumannii* BM4454, a low-level pan-aminoglycoside resistant clinical isolate (Magnet et al., 2001). The polycistronic *adeABC* transcript was confirmed experimentally to encode AdeA (MFP), AdeB (RND transporter), and AdeC (OMF; Magnet et al., 2001; Marchand et al., 2004). Because the substrate specificity of AdeAB is very similar to that of MexXY, we have no doubt that AdeAB is a functional homolog of MexXY in *A. baumannii*. AdeC is not essential for AdeAB-mediated resistance (Marchand et al., 2004), indicating that AdeAB recruits another outer membrane protein to form a functional tripartite complex, as observed for the MexXY pump with OprM in *P. aeruginosa* (Aires et al., 1999; Mine et al., 1999). We do not rule out the possibility that AdeAB is functional with AdeC, as observed for the MexXY pump with its linked outer-membrane channel OprA, which was not essential in *P. aeruginosa* PA7 (Morita et al., 2012). The *adeAB* genes are usually present, but the *adeC* gene was not found in ~40% of clinical isolates (Nemec et al., 2007). Our phylogenetic analysis showed that AdeC is more closely related to OprM and OprJ than to OprA in the OprM outer membrane family of *P. aeruginosa* (**Figure 2**).

The *adeABC* operon is expressed at low levels in natural isolates of *A. baumannii* due to stringent control by the AdeRS two-component system, which is encoded adjacent to *adeABC*, but transcribed in the opposite direction (Marchand et al., 2004; Table 1). Mutations (e.g., AdeR_Pro116Leu_, AdeS_Thr153Met_, or AdeS_Gly30Asp_) in AdeRS have been shown to be responsible for the constitutive expression of AdeABC (Marchand et al., 2004), which reminds us that mutations (e.g., ParR_Met59Ile_) in the ParRS two-component system are responsible for the constitutive expression of MexXY in *P. aeruginosa* (Muller et al., 2011). Overexpression of the AdeABC system in a tigecycline non-susceptible clinical isolate was due to the transposition of a copy of IS*Aba1* into *adeS* (Ruzin et al., 2007). Very recently, a truncated AdeS kinase protein generated by an IS*Aba1* insertion was shown to be correlated with enhanced* adeABC *expression in *A. baumannii* (Sun et al., 2012). Other regulatory mechanism(s) were shown to be involved in *adeABC *overexpression without any previously known mutation (Sun et al., 2010). Recently the AdeABC ortholog was shown to be a contributor to multiple antimicrobials, including aminoglycosides, in *Acinetobacter *genomospecies 13TU, a non-*A. baumannii *species (Roca et al., 2011).

## FUTURE PERSPECTIVES

MexXY is one of the potential targets for novel anti-pseudomonas agents. Its inhibitor is able to not only potentiate previously used ineffective antimicrobial agents (e.g., aminoglycosides against aminoglycoside-resistant *P. aeruginosa* and *B. cepacia *complex), but also to speed up the development of novel anti-pseudomonas agents. Because there are a significant number of potential drug targets encoded by the genome of *P. aeruginosa* (e.g., products of essential genes; Morita et al., 2010), it is the most promising therapeutic strategy to conquer the impermeability barriers of these bacteria. The efflux inhibitor MP 601384, which has specificity toward aminoglycoside-accommodating RND efflux systems and is not toxic to bacteria, is the only MexXY inhibitor reported so far (Jassem et al., 2011). Uncultured bacteria and plants are predicted to be a significant reservoir of novel antimicrobial agents (Stavri et al., 2007; Piel, 2011). Screening novel antibacterial agents, including a MexXY inhibitor, is currently in progress in our laboratory (e.g., Shiota et al., 2004).

## Conflict of Interest Statement

The authors declare that the research was conducted in the absence of any commercial or financial relationships that could be construed as a potential conflict of interest.
